# Characterization of Silver Nanoparticles Internalized by *Arabidopsis* Plants Using Single Particle ICP-MS Analysis

**DOI:** 10.3389/fpls.2016.00032

**Published:** 2016-02-01

**Authors:** Dongping Bao, Zhen Guo Oh, Zhong Chen

**Affiliations:** ^1^Natural Sciences and Science Education, National Institute of Education, Nanyang Technological UniversitySingapore, Singapore; ^2^School of Biological Sciences, Nanyang Technological UniversitySingapore, Singapore

**Keywords:** silver nanoparticles, *Arabidopsis thaliana*, enzymatic digestion, transport, SP-ICP-MS, TEM, biotransformation

## Abstract

Plants act as a crucial interface between humans and their environment. The wide use of nanoparticles (NPs) has raised great concerns about their potential impacts on crop health and food safety, leading to an emerging research theme about the interaction between plants and NPs. However, up to this day even the basic issues concerning the eventual fate and characteristics of NPs after internalization are not clearly delineated due to the lack of a well-established technique for the quantitative analysis of NPs in plant tissues. We endeavored to combine a quantitative approach for NP analysis in plant tissues with TEM to localize the NPs. After using an enzymatic digestion to release the NPs from plant matrices, single particle-inductively coupled plasma-mass spectrometry (SP-ICP-MS) is employed to determine the size distribution of silver nanoparticles (Ag NPs) in tissues of the model plant *Arabidopsis thaliana* after exposure to 10 nm Ag NPs. Our results show that Macerozyme R-10 treatment can release Ag NPs from *Arabidopsis* plants without changing the size of Ag NPs. The characteristics of Ag NPs obtained by SP-ICP-MS in both roots and shoots are in agreement with our transmission electron micrographs, demonstrating that the combination of an enzymatic digestion procedure with SP-ICP-MS is a powerful technique for quantitative determination of NPs in plant tissues. Our data reveal that Ag NPs tend to accumulate predominantly in the apoplast of root tissues whereby a minor portion is transported to shoot tissues. Furthermore, the fact that the measured size distribution of Ag NPs in plant tissue is centered at around 20.70 nm, which is larger than the initial 12.84 nm NP diameter, strongly implies that many internalized Ag NPs do not exist as intact individual particles anymore but are aggregated and/or biotransformed in the plant instead.

## Introduction

The increasing use of nano-enabled products in our society inevitably leads to more release of engineered nanoparticles (NPs) into the environment. In the agriculture sector for instance, silver nanoparticles (Ag NPs) are often incorporated into nano-agrochemicals, nano-biosensors, and used for nano-agri-food production (Sekhon, 2014). Accumulating evidence demonstrating the entry of these NPs into plant tissues raises great concern about their potential impact on crop health and food safety (Lee et al., 2008; Ma et al., 2010; Geisler-Lee et al., 2012; Wang et al., 2012; Zhao et al., 2013). Plant uptake of NPs begins with NPs coming into contact with the semi-permeable cell wall which acts as a sieve with nano-sized pores, selectively restricting the passage of foreign particles into the plant cell (Knox, 1995; Fleischer et al., 1999; Moore, 2006; Navarro et al., 2008). Currently, studies on the interaction of NPs with plants focusing on the phytotoxicity and accumulation precede the actual quantification of NPs in plant tissues, of which the latter is of vital importance for environmental and food risk assessment.

The primary challenge to study nanomaterials in plants lies in the detection of internalized NPs within plant tissue matrices and the measurement of their specific characteristics, such as particle size, number, size distribution, and concentration. Several techniques have been explored for the detection of NPs, including Raman spectroscopy, X-Ray absorption spectroscopy, and transmission electron microscopy (TEM) (Ostrowski et al., 2015), but unfortunately all of those techniques have certain limitations. Impurities or natural occurring plant composites could overwhelm the unique signatures of NPs or, in the situation where only a small portion of the tissues is being analyzed, the outcome may not be representative for the whole plant (Kole et al., 2013; Dan et al., 2015; Ostrowski et al., 2015).

Single particle-inductively coupled plasma-mass spectrometry (SP-ICP-MS) was based on the well-established ICP-MS technique and has proven to be a powerful tool to directly quantify single particle size, concentration, and size distribution (Degueldre et al., 2006; Tuoriniemi et al., 2012; Loeschner et al., 2013; Peters et al., 2014). Dan et al. (2015) have successfully used SP-ICP-MS for the measurement of the size distribution of gold nanoparticles (Au NPs) in NP-exposed tomato plants. The SP-ICP-MS method basically utilizes a mass spectrometer to detect the pulse (non-continuous) signals that are generated by each NP entering the plasma (Laborda et al., 2011; Mitrano et al., 2012). Both particle size and particle concentration affect such signals in intensity and in frequency, respectively, making SP-ICP-MS a sensitive technique that is useful for the analysis of NPs at low concentrations (Degueldre and Favarger, 2003; Degueldre et al., 2006; Mitrano et al., 2014a).

Single particle-inductively coupled plasma-mass spectrometry has been applied in the analysis of water, food matrices, materials, biological tissues, body fluids for trace metals, and toxic elements. Among all those tests it is the biological tissues that challenge the accuracy of SP-ICP-MS measurements most. The complex matrices require the use of a strong acid extraction procedure to release the NPs from the matrix, a procedure that could affect these NPs chemically. This problem could be solved by using a macerating enzyme digestion method (Marshall et al., 2007). Dan et al. (2015) reported high recoveries of Au NPs when using such a special macerating enzyme that appeared to release the NPs from plant tissue without changing the size distribution of the NPs.

We have used the same macerating enzyme for the release of Ag NPs with the model plant species *Arabidopsis thaliana* (*Arabidopsis*) exposed to 10 nm Ag NPs. The characteristics of the internalized Ag NPs were determined with SP-ICP-MS. Localization of the NPs in plant tissues at subcellular level was examined by conventional TEM. Our goal is to establish a reliable method to determine Ag NPs uptake and accumulation in *Arabidopsis* tissues. Moreover, we aim to depict a deposition pattern of Ag NPs in plant tissues and to examine possible translocation of Ag NPs toward the aerial part of the plant.

## Materials and Methods

### Germination and Growth Conditions

*Arabidopsis thaliana* Columbia (Col) wildtype seeds were surface-sterilized with 75% ethanol and 15% clorox followed by thrice washing using deionized water. After washing, 30 sterilized seeds were plated on 1/2 MS (Murashige and Skoog, Duchefa) medium supplemented with 1% sucrose and stratified at 4°C for 2 days in the dark. Seeds were germinated and grown in a nearly vertical position at 22°C with a 12 h/12 h light/dark regime and a light intensity of 120 μmol m^-2^ s^-1^ for 2 weeks before the seedlings were being subjected to NPs exposure.

### Nanoparticle Treatment and Enzymatic Digestion

Silver (Ag) NPs (Cat #730785) with an average particle size of 10 nm were purchased from Sigma–Aldrich (USA) and stabilized in sodium citrate. The NPs were sonicated for 30 min at 37 kHz using an ultrasonic cleaner to aid homogenous suspension and reduction of aggregation. Dilutions were prepared using deionized water and subsequently filtered with 0.2 μm sterilized filter before application. The NP treatment was performed by transferring 2-week-old *Arabidopsis* seedlings to 1/2 MS media containing 0.02 mg/L Ag NPs which was mixed with the medium before solidification. After growing for 2 weeks, whole plants were sacrificed and separated into shoot and root tissues. These tissues were washed thrice with deionized water before homogenization by a hand-held tissue homogenizer in 2 mM citrate buffer, with the pH adjusted optimally in the range of 3.5–7.0 for Macerozyme R-10 in accordance with the manufacturer’s instructions. After homogenization, samples were treated with 5% Macerozyme R-10 and shaken in a 37°C incubator for 24 h. After digestion, the samples were settled and diluted with ultrapure water for SP-ICP-MS analysis.

### Effects of Macerozyme R-10 on Ag NPs

Macerozyme R-10 (Yakult, Japan) is a complex macerating enzyme from *Rhizopus* sp. containing cellulase, hemicellulase, and pectinase which enables it to digest plant tissues and liberate the internalized NPs. In this study, Macerozyme R-10 was first investigated for its effect on Ag NPs such as dissolution and/or aggregation. This was performed as described by Dan et al. (2015). In short, 10 nm Ag NPs standard was diluted to 0.02 mg/L in 5% enzyme solution and homogenized with a hand-held tissue homogenizer. The samples were then shaken at 37°C for 24 h in an incubator. Thereafter, they were settled and diluted with ultrapure water for SP-ICP-MS analysis.

### SP-ICP-MS analysis

All samples were analyzed using a PerkinElmer NexION 300S ICP-MS operated in the single particle mode (PerkinElmer, 2015). Instrumental conditions were optimized for maximum sensitivity for ^107^Ag. The 30 nm Au NP standard was used for particle calibration to measure the particle size of Ag NPs in the samples and to determine the transport efficiency. This standard was also used at different concentrations to derive the detection limit. The detection limit was defined as the minimum detectable size of a single NP (Lee et al., 2014), which was 10 nm in this analysis. The settling time was eliminated to make continuous measurements possible whereas the dwell time was set to 50 μs. These analytical parameters have been evaluated and chosen for collection of metallic NP event data (Gray et al., 2014; Hineman and Stephan, 2014). The sampling time was set to 30 s. Syngistix software with the Nano Application Module was used for data collection, processing, and statistical analysis at PerkinElmer.

### Detection of Ag NPs Translocation by Transmission Electron Microscopy (TEM)

Roots and leaves were sampled from the plants dosed with Ag NPs grown on 1/2 MS plate as described above. Four-week-old root and leaf tissues were pre-fixed using 2% paraformaldehyde and 2% glutaraldehyde in 0.05 M sodium cacodylate buffer at pH 7.2 for 4 h at 4°C. After dehydration through a series of ethanol, the specimen was infiltrated, fixed in spurr resin and polymerized for 24 h at 70°C. Ultrathin sections were made on a Leica Ultracut UCT ultra-microtome and were stained thereafter with 2% uranyl acetate and Reynolds lead citrate. TEM images were obtained using a Jeol JEM-1230 (Japan) transmission electron microscope at an accelerating voltage of 80 kV.

### Image Processing

For the image editing and composition of the figure we used the open source programs GNU Image Manipulation Program (GIMP) and Inkscape.

## Results

### Effect of Macerozyme R-10 on Ag NPs

**FIGURE 1:**
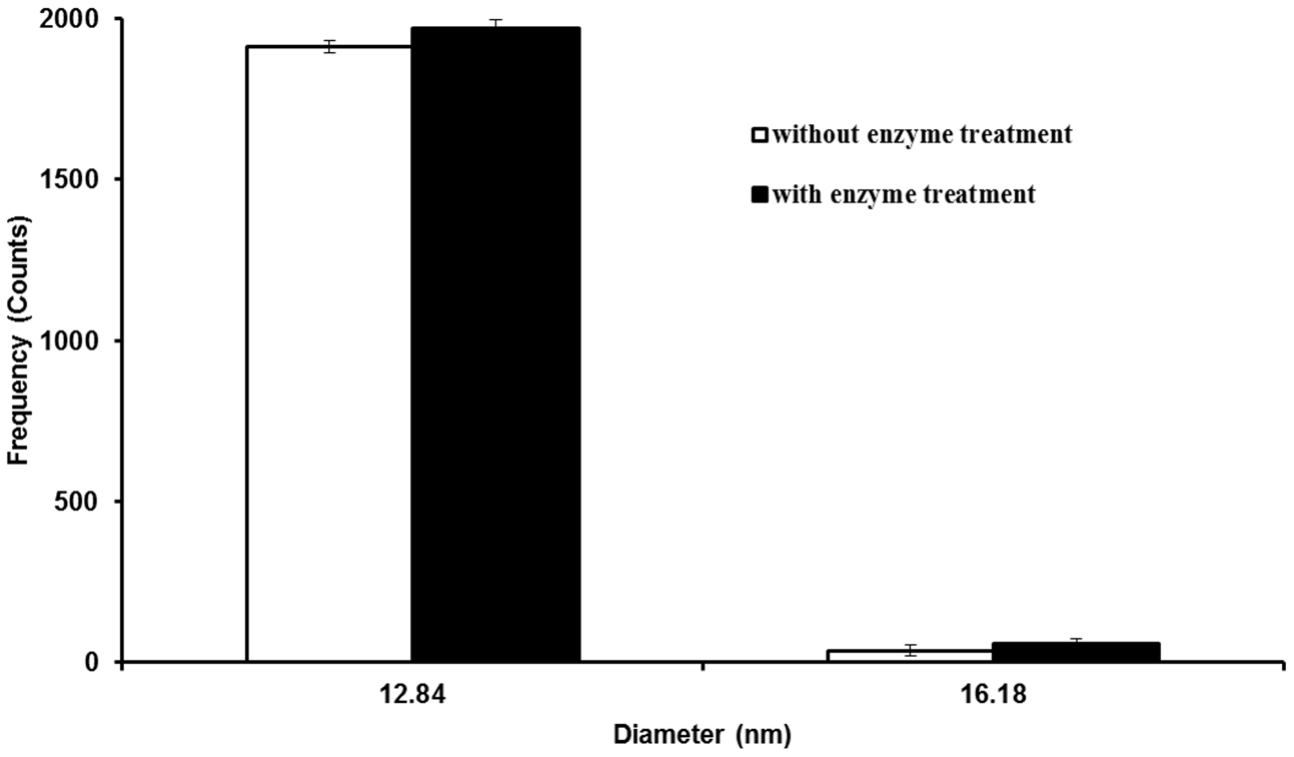
**Particle size distribution histogram of 10 nm silver nanoparticles (Ag NPs) with and without Macerozyme R-10 treatment.** Error bars indicate standard deviations from two independent experiments.

To investigate the effect of Macerozyme R-10 on Ag NPs during plant tissue digestion, Ag NPs were treated with Macerozyme R-10 and analyzed using SP-ICP-MS. Based on the sizes of Ag NPs detected, the particle size distribution histogram was generated. **Figure 1** shows the number of Ag counts in different sizes for Ag NPs that were subjected to Macerozyme R-10 and the non-treated control sample. The most frequent (>97% of the total counts) size detected had a measured diameter of approximately 12.84 nm, slightly larger than the 10 nm NP size the manufacturer had described. This slight increase in size could be explained by the innate size of the Ag NP tested, because there was no significant difference in particle size distribution between enzyme-treated and non-treated: the number of counted NPs that were detected at 12.84 nm diameter did not differ significantly (**Figure 1**). These results demonstrated that application of Macerozyme R-10 causes no measurable effect (dissolution or aggregation) on the size distribution of Ag NPs as determined by SP-ICP-MS, which classifies it as a suitable enzyme for extraction of Ag NPs from plant tissues in this study.

### Accumulation of Ag NPs in Root and Shoot Tissues and SP-ICP-MS Analysis

**FIGURE 2:**
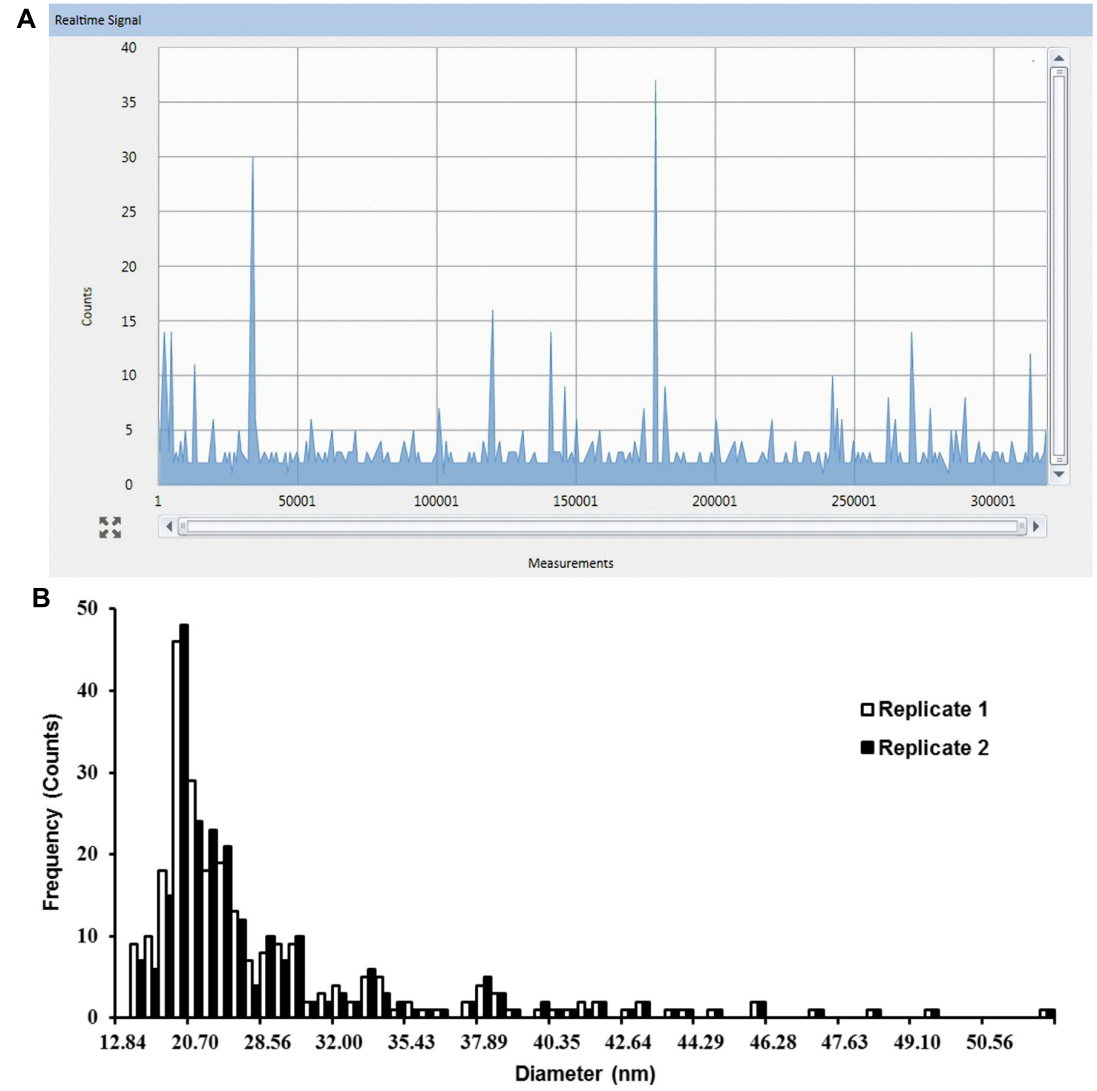
**Single particle-inductively coupled plasma-mass spectrometry (SP-ICP-MS) analysis of internalized Ag NPs in root tissues of *Arabidopsis* plants exposed to 0.02 mg/L 10 nm Ag NPs for 2 weeks. (A)** Raw counts for Ag NPs detected from replicate 1 and **(B)** derived size distribution histogram of Ag NPs for two biological replicates.

To determine the outcome of exposure to Ag NPs, Ag NPs treated plants were digested with Macerozyme R-10 prior to SP-ICP-MS analysis. **Figure 2A** shows the raw data from the enzymatic digested roots (Replicate 1) that were previously dosed with 0.02 mg/L 10 nm Ag NPs for 2 weeks. Typical NP pulse signals were observed. **Figure 2B** shows the resulting particle size distribution of released Ag NPs as processed from the data in **Figure 2A**. Two replicates yielded highly similar results, displaying a peak at around 20.70 nm (**Figure 2B**). As this observed NP size was larger than the initial dosed 12.84 nm, NPs may have been modified in the plant tissues.

**FIGURE 3:**
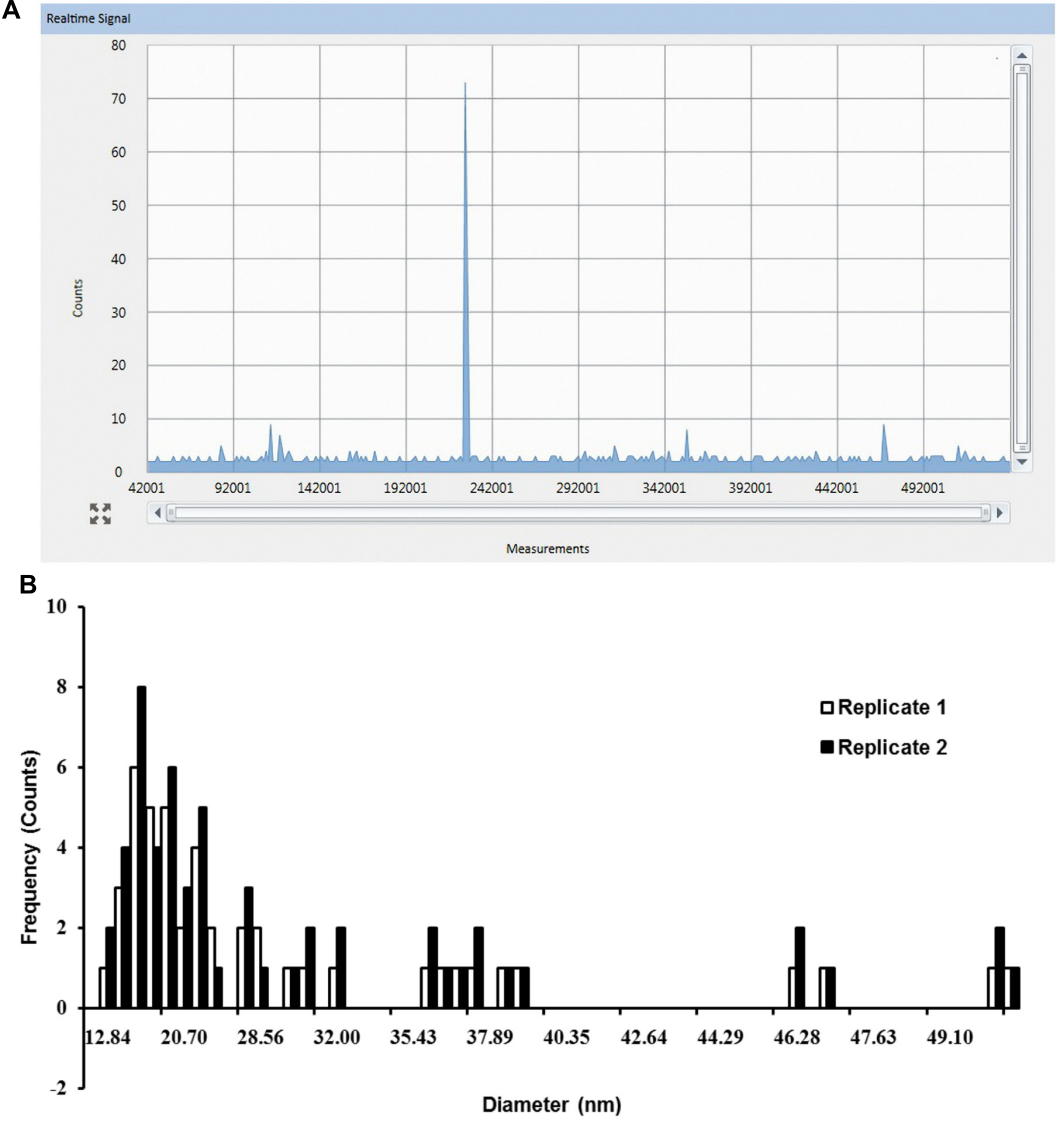
**SP-ICP-MS analysis of internalized Ag NPs in shoot tissues of *Arabidopsis* plants exposed to 0.02 mg/L 10 nm Ag NPs for 2 weeks. (A)** Raw counts for Ag NPs detected from replicate 1 and **(B)** derived size distribution histogram of Ag NPs for two biological replicates.

In the same experiment, SP-ICP-MS also detected Ag NPs signals in shoot tissues (**Figure 3A**) of *Arabidopsis* plants which grew on 1/2 MS medium containing 0.02 mg/L 10 nm Ag NPs. Compared to the root (**Figure 2A**), fewer pulse signals (**Figure 3A**) were detected in the shoot, indicating only a minor presence of Ag NPs in *Arabidopsis* shoot tissues. A nearly constant background signal at a level of circa 2 counts was observed in Syngistix (**Figures 2A and 3A**). These continuous background signals indicate the presence of dissolved Ag in the solution (Gray et al., 2014; Mitrano et al., 2014b). The corresponding particle size distribution of Ag NPs (**Figure 3B**) was processed from the data in **Figure 3A**. Similarly, particle size distribution in shoots also centered at 20.70 nm. Note that the particle size distribution in shoots showed less uniformity than in roots, as reflected by the large difference between the two replicates. The difference in counts between the root and shoot is striking, as root tissues had a five times higher reading frequency than shoot tissues (**Figures 2B** and **3B**). This experiment not only establishes the uptake and accumulation of Ag NPs by *Arabidopsis* root, but also strongly suggests that a translocation of Ag NPs occurs toward the plant shoot. Furthermore, the majority of the peaks for both histograms appeared in between 12.84 and 28.56 nm. The Syngistix software tabulated the most frequent size detected for both histograms as 19–20 nm and the mean size detected as 26–27 nm, which was approximately 1.52- and 2.06-fold larger than the size of an individual Ag NP. These results indicate that 10 nm Ag NPs hardly exist as intact individual Ag NPs in plant tissues.

### Translocation of Ag NPs in *Arabidopsis* and TEM

**FIGURE 4:**
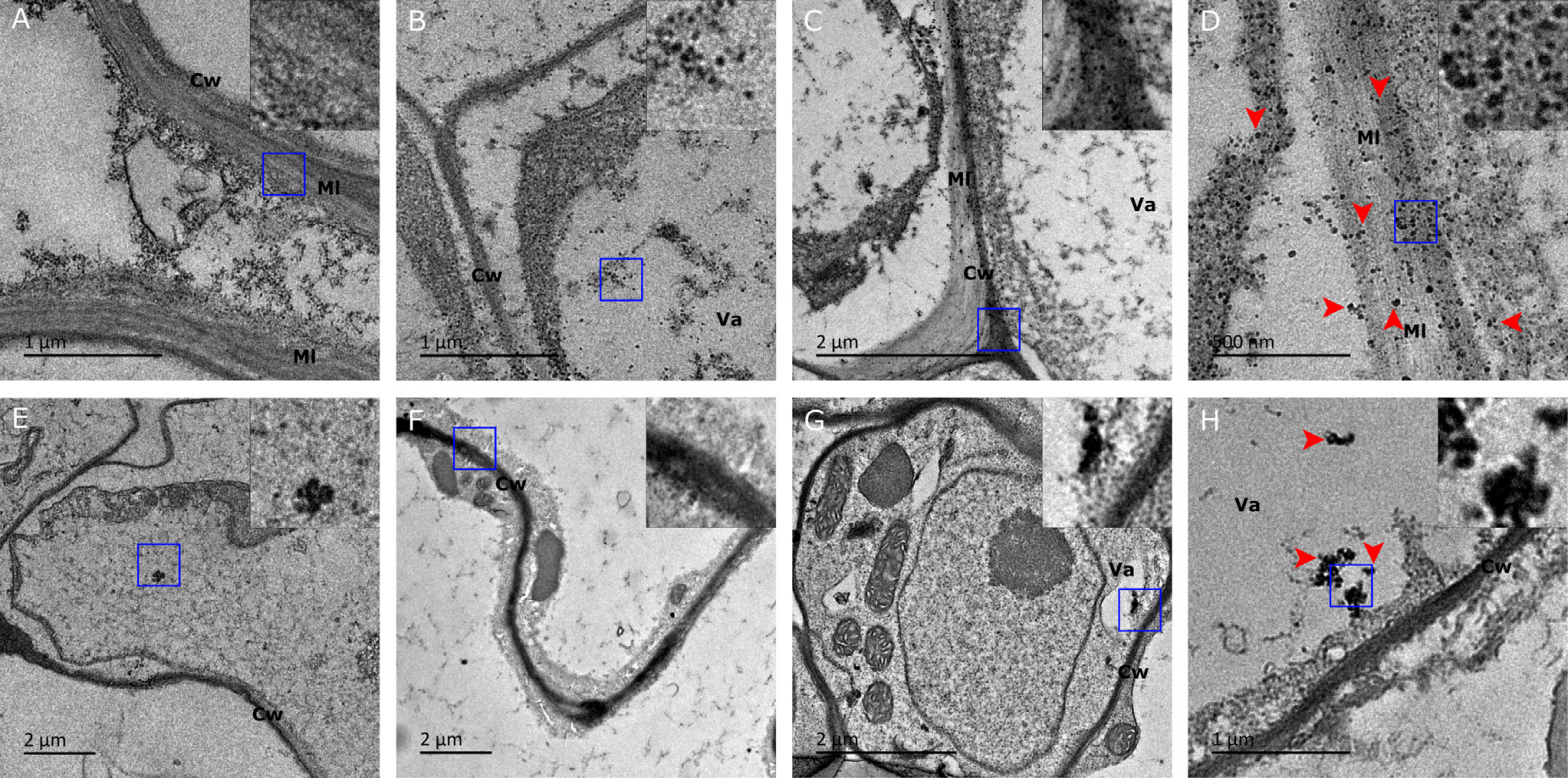
**Transmission electron microscopy (TEM) images of Ag NP localization in tissue of plants fed with 10 nm Ag NPs for 2 weeks.** Accumulation of Ag NPs in *Arabidopsis* roots (upper) and leaves (lower) were shown. **(A)** No Ag NPs visible in cell wall and middle lamella of a control cell. **(B)** Ag NPs accumulated in the vacuole, **(C)** the boundary area between two cells and **(D)** the vicinity of the cell wall and its middle lamella matrices. **(E)** No accumulation of NPs in a control leaf cell. **(F)** Ag NPs accumulated in the cell wall, **(G)** in the vacuole and **(H)** aggregated in a leaf cell. Note that image **(D)** is an enlargement of the central section of image **(C)**. The inserts show a three times higher magnification of the square area (blue) indicated in each panel. Red arrows were indicative of Ag NPs. Cw, cell wall; Va, vacuole; Ml, middle lamella.

To establish whether the shootward Ag NPs translocation occurs as suggested by SP-ICP-MS analysis, we examined both root and leaf tissues using TEM to directly visualize the localization of Ag NPs at subcellular level. Micrographs revealed that electron-dense particles were predominantly present in the root as shown by numerous free NPs and some aggregated clumps in root cells (**Figures 4B–D**). Root-fed Ag NPs shown as individual particles and aggregated clumps were observed all over the cell wall, middle lamella plasma, intercellular space, and in the vacuole, demonstrating massive accumulation and internalization of Ag NPs in *Arabidopsis* root (**Figures 4B–D**). The aggregated clumps could often be found in the middle lamella and the cell wall. We could not observe these universally scattered dark particles in the untreated control root cells whilst we did visualize the same kind of background dots there (**Figure 4A**). Furthermore, in the cell wall and its middle lamella we observed only few black spots compared to the NP-fed roots where NPs were obviously retained across the cell wall, probably during the internalization process (**Figure 4D**). We conclude that both the cell wall and the middle lamella were the primary accumulation sites for NPs.

In the leaf of the same plant, deposition of Ag NPs was also observed around the cell wall and in the vacuole (**Figures 4F–H**). Ag NPs seemed to be prone to forming aggregates, resulting in varied size of clusters (**Figure 4H**). In the absence of NPs, dark specks could be observed at several places in the leaf cell micrographs (**Figure 4E**). These specks appeared to have a lighter density though, unlike the stodgy, much darker and rather compact particles that are present in NP-treated plants (**Figure 4** inserts). The difference between the specks in untreated control plants and the dense particles in NP-treated plants are especially telling when examined under larger magnification (**Figure 4** inserts). Therefore, it is believed that only the latter, darker particles represent the NP agglomerates. It is worth noting that, compared to the vast presence of Ag NPs in many root cells, Ag NPs could only be detected in some of the leaf cells, thereby establishing that silver NPs translocation from root to leaf did occur *in vivo*, albeit at a small scale only. Our TEM analysis agreed well with the SP-ICP-MS results that had demonstrated that the NPs that had accumulated in leaf tissues showed large variation in size and relatively few readings compared to NPs in root tissues.

## Discussion

We have described the combination of an enzymatic digestion method with SP-ICP-MS technique for the determination of size and size distribution of internalized Ag NPs in *Arabidopsis* plants. Our study expands the utilization of the analytical method developed by Dan et al. (2015) in characterizing foreign NPs bioaccumulated in plants. TEM enabled us to examine their localization in plant tissues at subcellular level.

Application of Macerozyme R-10 does not change the size of Ag NPs in SP-ICP-MS analysis (**Figure 1**), in the sense that enzymatic treatment caused neither dissolution nor aggregation of Ag NPs in our experiment. Macerozyme R-10 treatment can release Ag NPs from *Arabidopsis* plants, which makes it suitable for the subsequent SP-ICP-MS analysis.

One of the advantages of the SP-ICP-MS technique is its capability to differentiate between particles and dissolved metal concentration in the measurement. The detected pulses (on top of the baseline) represent individual particle events and the particle mass is determined by the intensity of the ICP-MS response. A continuous response, on the other hand, indicates the presence of dissolved metal in the solution (Hineman and Stephan, 2014). A proper choice of the dwell time window is critical here, because the duration of the dwell time has effect on the level of background signal present in the measurement and will affect the counting and sizing of NPs (Hineman and Stephan, 2014). In **Figures 2A** and **3A** continuous signals in the background were observed, which suggests the presence of dissolved Ag^+^ in the samples, resulting in a slightly elevated baseline. The dissolved Ag^+^ concentration seems to be constant in root and shoot samples under the condition that we used. By subtracting the background response (the baseline) from the NP response in single particle mode in Syngistix, an accurate NP mass determination can be achieved, given the relatively low particle number concentrations used (Hineman and Stephan, 2014; Mitrano et al., 2014b).

The micrographs of the root sections clearly show that the cell wall and the middle lamella act as primary accumulation sites for NPs. It is at this location that we find the highest number of NPs accumulated. Although we are not sure whether subsequent symplastic transport occurs, the abundance of NPs inside the cell (e.g., vacuole) and at the cell junction area where the cell wall connects to more than one cell (**Figures 4C,D**) suggests the intercellular passage of NPs through the cell wall. The existence of such a NP translocation pathway was reported earlier by Geisler-Lee et al. (2012), when they described apoplastical transport of Ag NP after direct uptake by *Arabidopsis* roots.

Earlier observations indicated that soil-grown *Arabidopsis* roots treated with 20 nm Ag NPs may bio-accumulate up to 10 times more NPs than shoots (Geisler-Lee et al., 2014). The fact that in our experiment the Ag NP counts in root tissues were five times as high as in shoot tissues (**Figures 2** and **3**) strongly indicates the predominant accumulation of Ag NPs in the root with only a minor translocation of Ag NPs toward the shoot. This is in line with our TEM analysis that showed more Ag NPs in root tissue than in shoot tissue (**Figure 4**). Micrographs reveal that NPs are widespread as monodisperse particles in many root cells, whereas variously sized NP aggregates were observed in a small portion of the leaf cells only, each aggregate displaying a distinct mass distribution and shape (**Figure 4H**). During the preparation of the specimen for TEM, artifacts like precipitates can occur, as can be observed in the controls (**Figures 4A,E**). For this reason, it is not possible to accurately pinpoint for an individual particle whether it is indeed a silver NP that has accumulated in the tissues. On the other hand, we did notice specific differences in location and accumulation patterns as well as a striking density difference between NPs and precipitates (**Figure 4** inserts). Together with the SP-ICP-MS quantification data for *Arabidopsis* root and shoot, our results strongly indicate the presence of Ag NPs within these plant tissues.

Other NPs such as titanium dioxide NPs and Au NPs have also been found to be transported toward the shoot area (Kurepa et al., 2010; Dan et al., 2015). It is interesting to note that Taylor et al. (2014) found contradictory results with alfalfa, namely that there was no uptake of Au NPs. In barley plants on the other hand, uptake of Au NPs does take place as Au NPs accumulate in the roots whilst no transfer of Au NPs into the leaves can be observed (Feichtmeier et al., 2015). Smaller Au NPs (10 nm) than Dan et al. (2015) used in tomato (40 nm) have been reported to fail to pass through algal cell walls (Proseus and Boyer, 2005). These rather divers results indicate that the processes of NP uptake and translocation are far from simple. Whether uptake or translocation happens will not only be determined by the chemical nature of the NPs used and the diameter of the NPs but also depend on specific biological aspects of the plants that are used. We were not able to envisage a NP shootward translocation pathway in *Arabidopsis* in this study.

From the SP-ICP-MS analysis, it can be concluded that the most frequently detected diameter of Ag NPs is 19–20 nm and the average diameter of Ag NPs detected is 26–27 nm, which is approximately 1.52 and 2.06 times larger than the initial diameter (12.84 nm) of monodisperse Ag NPs. These results strongly point toward possible transformations and/or aggregation of Ag NPs during or after their internalization. Whether they are modified in the cytoplasm itself or on their way before or during the entering of the cell is currently unknown. Interestingly, Dan et al. (2015) found no alterations using Au NPs (40 nm), these NPs appeared to remain intact after internalization by tomato plants. An easy explanation for this NP alteration discrepancy can be found in the differences between the properties of the NPs that were used, as it is well known that Au NPs are chemically more stable than Ag NPs (Kuzma et al., 2012). Alternatively, this discrepancy can be explained by the difference in biological context (e.g., cell walls, intercellular space, and fluid cytoplasm) when using plants as different as *Arabidopsis* and tomato. Thus, similar types of NPs may behave differently in the process of internalization for different plants since the uptake is likely species-dependent, probably due to different properties of the cell wall and cytoplasm.

Using SP-ICP-MS in combination with TEM, we succeeded in combining a quantitative characterization of Ag NPs with visual NP localization in plant tissues, which enabled us to clarify where, and to what extent, NPs would be translocated in *Arabidopsis*. We found that in Ag NP treated plants the NPs accumulate predominantly at the middle lamella and cell walls in root tissue and that some Ag NPs can be translocated toward the leaves. The true nature of inter- and intracellular NP transport is not known yet nor do we know which transformations happen to the Ag NPs in plant tissues. The next challenge will be to bring light into the uptake and transport pathways and to unravel their underlying mechanisms.

## Author Contributions

ZC initiated the project. DB did the TEM and data analysis. ZGO performed SP-ICP-MS. DB, ZGO, and ZC wrote the manuscript.

## Conflict of Interest Statement

The authors declare that the research was conducted in the absence of any commercial or financial relationships that could be construed as a potential conflict of interest.
